# Physical Activity Alters Functional Connectivity of Orbitofrontal Cortex Subdivisions in Healthy Young Adults: A Longitudinal fMRI Study

**DOI:** 10.3390/healthcare11050689

**Published:** 2023-02-26

**Authors:** Jannik Claus, Neeraj Upadhyay, Angelika Maurer, Julian Klein, Lukas Scheef, Marcel Daamen, Jason Anthony Martin, Rüdiger Stirnberg, Alexander Radbruch, Ulrike Attenberger, Tony Stöcker, Henning Boecker

**Affiliations:** 1 Clinical Functional Imaging Lab, Department of Diagnostic and Interventional Radiology, University Hospital Bonn, Venusberg-Campus 1, 53127 Bonn, Germany; 2 German Center for Neurodegenerative Diseases, Venusberg-Campus 1, Building 99, 53127 Bonn, Germany; 3 Department of Neuroradiology, University Hospital Bonn, Venusberg-Campus 1, 53127 Bonn, Germany; 4 Department of Diagnostic and Interventional Radiology, University Hospital Bonn, Venusberg-Campus 1, 53127 Bonn, Germany

**Keywords:** exercise, functional connectivity, mood, orbitofrontal cortex, physical activity, rsfMRI

## Abstract

Physical activity (PA) plays an important role in affect processing. Studies describe the orbitofrontal cortex (OFC) as a major hub for emotion processing and the pathophysiology of affective disorders. Subregions of the OFC show diverse functional connectivity (FC) topographies, but the effect of chronic PA on subregional OFC FC still lacks scientific understanding. Therefore, we aimed at investigating the effects of regular PA on the FC topographies of OFC subregions in healthy individuals within a longitudinal randomized controlled exercise study. Participants (age: 18–35 years) were randomly assigned to either an intervention group (IG; N = 18) or a control group (CG; N = 10). Fitness assessments, mood questionnaires, and resting state functional magnetic resonance imaging (rsfMRI) were performed four times over the duration of 6 months. Using a detailed parcellation of the OFC, we created subregional FC topography maps at each time point and applied a linear mixed model to assess the effects of regular PA. The posterior–lateral right OFC showed a group and time interaction, revealing decreased FC with the left dorsolateral prefrontal cortex in the IG, while FC in the CG increased. Group and time interaction in the anterior–lateral right OFC with the right middle frontal gyrus was driven by increased FC in the IG. The posterior–lateral left OFC showed a group and time interaction based on differential change in FC to the left postcentral gyrus and the right occipital gyrus. This study emphasized regionally distinctive FC changes induced by PA within the lateral OFC territory, while providing aspects for further research.

## 1. Introduction

The orbitofrontal cortex (OFC) plays a key role in emotion, the processing of reward value, and response inhibition [1]. It is the part of the prefrontal cortex that has significantly expanded in recent evolution and has neuronal connections to many parts of the brain that process emotion and executive function, i.e., sensory cortices, amygdala, prefrontal cortex, cingulate cortex, striatum, and hypothalamus [2]. In recent years, researchers have tried to fill in the gap of explaining the role of this poorly understood brain region. Previous studies have shown that damage to the OFC impacts emotion, social behavior, and cognitive control [3,4,5]. Altered brain activity and/or functional connectivity (FC) of the OFC have been observed in different psychiatric disorders [6]. Notably, studies have shown that patients with major depression (MD) have altered FC of the OFC [7,8]. Frodl et al. [8] reported reduced FC of the OFC to the precuneus, dorsal anterior cingulate cortex, and cerebellum, as well as an increased FC from the OFC to left motor areas, right inferior frontal operculum, and right dorsolateral prefrontal cortex (DLPFC).

The current literature mostly divides the OFC into two functional parts: the medial and lateral OFC [2]. Studies assessing OFC FC reported that the medial part is preferentially involved in representing reward value (affective value), while the lateral part represents punishment and is active when not receiving an expected reward (non-reward) [2,9,10]. Beyond this hedonic medial–lateral distinction, there is evidence for more fine-grained functional specializations within OFC subregions: For example, Kahnt et al. [11] proposed a subdivision of the OFC into six different areas based on their differential resting state FC profiles with the rest of the brain: medial (1), postcentral (2), central (3), and lateral areas (4–6). A similar parcellation was recently suggested by Du et al. [12]. These connectivity differences may not only reflect varying involvement in normal affective processing networks, but also a differential involvement in the development of depression and other psychiatric disorders.

There is an increasing interest in non-pharmacological strategies for psychiatric treatment. For symptomatic relief in psychiatric disorders, recent evidence indicates that various behavioral interventions, such as yoga [13], dancing [14], and also physical exercise (e.g., [15,16]) can support symptom relief in various psychiatric disorders. Physical activity (PA) has also been suggested to modulate the reward system of the human brain [17,18,19]. It has been well documented that psychiatric disorders and mood in general are influenced by exercise [17,20,21,22]. There is metanalytic evidence suggesting PA to be effective as additional treatment for affective disorders [23,24]. It has also been reported that regular exercise may prevent depression [24,25]. A recent review proposed differential FC characteristics of the OFC in affect processing [2]. However, there is a lack of understanding how regular exercise interventions influence the FC of different OFC regions and their role in affect processing in healthy subjects.

Therefore, we aimed at characterizing the FC changes in the subdivisions of the OFC [11] using whole-brain resting state functional magnetic resonance imaging (rsfMRI) data acquired at four time points over a longitudinal 6-month exercise intervention in healthy young adults. In addition, we were also interested in exploring how these diverse OFC FC changes are related to affective measures.

## 2. Materials and Methods

We conducted a longitudinal randomized controlled study (“RUNSTUD”; trial registration: Deutsches Register Klinischer Studien DRKS0021460) to examine the effects of PA over the course of 6 months. Participants were randomly assigned to either an intervention group (IG), with regular interval running sessions, or a control group (CG) and regularly underwent fitness assessments and affect-related questionnaires, as well as rsfMRI.

### 2.1. Participants

Participants of the study were healthy men or woman aged 18–35 who had not performed regular exercise in the past two years prior to the study. Recruiting was performed via social media and flyers handed out at the local university. Further inclusion criteria were right-handedness, being fluent in the German language, and no history as a professional athlete. Exclusion criteria were oncological, neurological, psychiatric, and cardiological diseases and substance abuse, as well as common MRI contraindications.

The descriptions of the sample selection and randomization were reported earlier [26,27] and are only summarized here. From 57 people originally enrolled in the study, N = 9 dropped out already during the pre-randomization basic examinations (see Figure S1 [26]). The remaining 48 subjects were randomized and allocated into the control group (CG, N = 16) and intervention group (IG, N = 32), with N = 27 IG and N = 15 CG participants starting the treatment and N = 6 IG and N = 4 CG, respectively, dropping out during the course of the intervention phase due to various reasons (including loss of interest or time constraints and injuries due to private activities). During the analysis, N = 1 participant from each group was excluded due to the development of clinical depression levels during the study’s course, while N = 1 IG participant completed the study, but only performed a low number of regular trainings due to illness, and N = 1 IG participant, because 3T MR data were not available. This left N = 18 participants in the IG and N = 10 in the CG for the analyses. For two subjects, the final T4 MR examination was not available: Here, only the completed examination days were considered in the longitudinal analyses.

The study was approved by the Ethics Committee of the Medical Faculty of the University of Bonn (No. 370/15). Participants were given an anamnestic questionnaire to make sure they met our inclusion and exclusion criteria. They were fully informed about the proceedings of the study, and written consent was acquired. A routine cardiological check-up was performed to make sure participants did not have any cardiac condition that would put them at risk. Further background characterization included a sociodemographic questionnaire, a vocabulary test [28], the Edinburgh-Handedness-Inventory [29], the Mini International Neuropsychiatric Interview (MINI, German Version 5.0.0) [30], the Beck Depression Inventory (BDI) [31], the Fagerström Test for Nicotine Dependence (FTND) [32], and a questionnaire for substance consumption. Before each MRI scan, female subjects were tested for pregnancy and all participants were screened again for MRI contraindications with written consent for the MRI acquired.

### 2.2. Experimental Procedure

Subjects were randomized into two groups in a 2:1 (IG:CG) ratio. At the beginning of the study (0 months, T0) and after 2 months (T2), 4 months (T4), and 6 months (T6), a graded exercise task [33] was performed on a treadmill (PPS S70, Woodway GmbH, Weil am Rhein, Germany). A more in-depth explanation of the parameters chosen in the RUNSTUD study was already published by our group [27]. The following parameters were recorded: heart rate (HR), oxygen uptake (VO2), and metabolic respiratory exchange ratio (RER) (Cortex meta-analyzer 3B, Leipzig, Germany, and Polar A360, Kempele, Finland). The performance index relVO2_max_ was calculated as the highest 30-second moving average of VO2 divided by body mass (mL/min/kg). Participants were also given a fitness tracker at the beginning of the study (Polar A360, Kempele, Finland) in order to assess the PA on an everyday basis. Members of the CG were instructed not to perform any regular exercise, but to maintain their current lifestyle. For participants in the IG, an individual fitness plan was created. This involved three interval running sessions per week lasting 25–45 min in total, aiming at 75–80% of their maximal heart rate. Furthermore, participants were not allowed to consume caffeine two hours prior to the fitness tests and were advised to restrain from heavy PA and alcohol consumption for 24 h. To assess the fitness of each individual accurately, regular fitness tests were scheduled every two months consecutively. After each examination, exercise plans were adjusted for every participant in the IG to their individual fitness. At each testing session, a set of questionnaires was completed by each participant in order to access his/her emotional state: The Positive and Negative Affect Schedule (PANAS) [34] and the state subscale of the State-Trait-Anxiety Inventory (STAI) [35]. The BDI was repeated to exclude participants who were exhibiting depressive symptoms.

The PANAS is a self-report questionnaire used to assess positive and negative affect with two separate subscales, each consisting of 10 items. The STAI examines anxiety levels both on the current (STAI state) and dispositional (STAI trait) level, using 20 items for each of the two subscales, with higher scores indicating greater anxiety levels (for more details, see [27]). While the baseline STAI trait was examined as a general background characteristic of the study participants, the STAI state values collected during each MR session were examined longitudinally (for details, see [27]).

### 2.3. Magnetic Resonance Imaging

#### 2.3.1. Data Acquisition

Resting state fMRI data were acquired using a 3T Siemens Magnetom Skyra MRI scanner with a 32-channel head-coil. The detailed protocol is characterized as follows: blood-oxygenation-level-dependent (BOLD) fMRI time-series were acquired using a 3D echo-planar imaging (EPI) sequence with repetition time (TR) = 570 ms, echo time (TE) = 30 ms, water-selective excitation flip angle = 15${}^{\circ }$ combined with spectral adiabatic fat inversion recovery, blipped-CAIPI 1x4z2 parallel imaging [36,37], phase partial Fourier factor 7/8, semi-elliptical sampling [38], 48 slices per slab, field-of-view = 192 × 192 × 144 mm, and voxel size = 3 × 3 × 3 mm. Moreover, complementary field map data were acquired for distortion correction. At the imaging sessions, we also acquired structural MRI data of every participant; the details are: TR = 2.5 s, TI = 1.1 s, TE = 5 ms, flip angle = 7${}^{\circ }$, 1x3z1 CAIPIRINHA parallel imaging and elliptical sampling [39], field-of-view = 192 × 192 × 144 mm, voxel size =1 × 1 × 1 mm, and total scan duration: 2 min 53 s.

#### 2.3.2. Data Analysis

Quality checks were performed using MRIQC [40]. Preprocessing of the rsfMRI data was performed using fmriprep 20.0.6 [41], which is based on Nipype 1.4.2 [42]. While full details on the preprocessing steps applied by fmriprep on the anatomical data can be found in Maurer et al. [27], for each functional BOLD run, the following preprocessing was included:

For every scan, the first 17 volumes were removed (as dummy scans). A reference volume and its skull-stripped version were generated using a custom methodology of fMRIPrep. A B0-nonuniformity map (or field map) was estimated based on a phase-difference map calculated with a dual-echo gradient-recall echo (GRE) sequence, processed with a custom workflow of SDCFlows inspired by the epidewarp.fsl script and further improvements in HCP Pipelines [43]. The field map was then co-registered to the target EPI reference run and converted to a displacements field map (amenable to registration tools such as ANTs [44]) (RRID:SCR_004757) with FSL’s fugue (FSL 5.0.9, RRID:SCR_002823) and other SDCflows tools. Based on the estimated susceptibility distortion, a corrected EPI reference was calculated for a more accurate co-registration with the anatomical reference. The BOLD reference was then co-registered to the T1w reference using bbregister (FreeSurfer, RRID:SCR_001847), which implements boundary-based registration [45]. Co-registration was configured with six degrees of freedom. Head-motion parameters with respect to the BOLD reference (transformation matrices and six corresponding rotation and translation parameters) were estimated before any spatiotemporal filtering using mcflirt (FSL 5.0.9) [46]. The BOLD time-series (including slice-timing correction when applied) were resampled onto their original native space by applying a single composite transform to correct for head motion and susceptibility distortions. These resampled BOLD time-series will be referred to as preprocessed BOLD in the original space, or just preprocessed BOLD. The BOLD time-series were resampled into the MNI152NLin2009cAsym space [47] (RRID:SCR_008796), generating a preprocessed BOLD run in the standard space. Several confounding time-series were calculated based on the preprocessed BOLD: framewise displacement (FD), DVARS, and three regionwise global signals. FD and DVARS were calculated for each functional run, both using their implementations in Nipype (following the definitions by Power et al. [48]). The three global signals were extracted within the cerebrospinal fluid (CSF), the white matter (WM), and the whole-brain masks. Additionally, a set of physiological regressors was extracted to allow for component-based noise correction (CompCor) [49]. Principal components were estimated after high-pass filtering the preprocessed BOLD time-series (using a discrete cosine filter with 128 s cut-off) for the two CompCor variants: temporal (tCompCor) and anatomical (aCompCor). For aCompCor, components were calculated within the intersection of a mask covering the subcortical regions and the union of CSF and WM masks calculated in the T1w space, after their projection to the native space of each functional run (using the inverse BOLD-to-T1w transformation). Components were also calculated separately within the WM and CSF masks. For each CompCor decomposition, the k components with the largest singular values were retained, such that the retained components’ time-series were sufficient to explain 50 percent of variance across the nuisance mask (CSF, WM, combined, or temporal). The remaining components were dropped from consideration. The head motion estimates calculated in the correction step were also placed within the corresponding confounds file. The confound time-series derived from head motion estimates and global signals were expanded with the inclusion of temporal derivatives and quadratic terms for each [50]. Frames that exceeded a threshold of 0.5 mm FD or 1.5 standardized DVARS were annotated as motion outliers. All resamplings can be performed with a single interpolation step by composing all the pertinent transformations (i.e., head motion transform matrices, susceptibility distortion correction when available, and co-registrations to anatomical and output spaces). Gridded (volumetric) resamplings were performed using antsApplyTransforms (ANTs), configured with Lanczos interpolation to minimize the smoothing effects of other kernels [51]. Non-gridded (surface) resamplings were performed using mri_vol2surf (FreeSurfer). This description was automatically generated by fmriprep (CC0 license) and slightly abbreviated by the authors of this paper.

Denoising of the preprocessed data was achieved with 3dTproject from the AFNI toolbox [52,53], by removing 12 motion parameters, as well as acompcor components obtained from the first 5 WM and 5 CSF parameters, generated by fmriprep, as explained above. This method has been found to be reliable in removing the effects of head motion, as well as cardiac and respiratory noise [54,55,56]. Afterwards, the data were smoothed with a 4 mm kernel size. For further processing, an average brain mask was computed by merging every subject’s brain mask into one and averaging the result. For the whole-brain analyses, a grey matter mask was generated by merging every subject’s grey matter mask, as computed by fmriprep, resampling it into the MNI152NLin2009cAsym space and thresholding the resulting mask to visually be a good representation of the average grey matter.

#### 2.3.3. Seed-to-Whole-Brain Analysis

**Figure 1:**
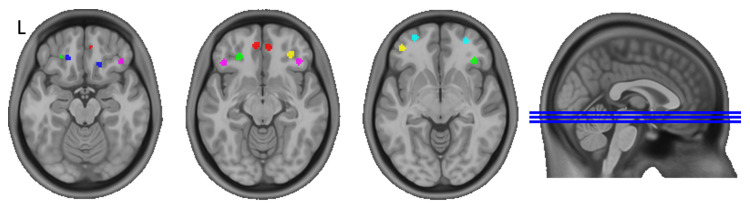
Spherical seeds (r = 4 mm) used for analysis. 1 = red, 2 = blue, 3 = green, 4 = violet, 5 = yellow, 6 = cyan. Separate seeds for left (L) and right (R) orbitofrontal cortex, respectively. Based on Kahnt et al. [11]. Overlayed on the MNI152NLin2009cAsym standard space template [47] using MRIcron [62].

Based on a detailed parcellation of the OFC by Kahnt et al. [11], small spherical regions of interest (ROIs) around the reported center coordinates (Table 1) were generated. As some studies reported differential functions of the left and right OFC [57,58], seeds were created separately for the left and right hemispheres (see Figure 1). We chose a radius of 4 mm, as a 5 mm radius resulted in multiple voxels overlapping and, therefore, being unsuitable for a differential analysis, although the smaller 4 mm was still sufficiently large for our data. Dual regression [59] was used to generate specific whole-brain regression maps for every region of interest. First, the time-series was extracted from each ROI using the regression model, and later, the extracted time-series were regressed against the whole-brain denoised rsfMRI data to obtain individual spatial maps corresponding to each ROI at each time point.

A well-known problem in fMRI studies of OFC function is the risk of image distortions and signal dropouts due to susceptibility effects [60,61]. In addition to off-line field-map-based distortion correction, we validated sufficient signal coverage for the OFC by creating FC topography maps of the bilateral OFC (see Figure S2).

### 2.4. Statistics

#### 2.4.1. Physiological Data

For the analysis of the physiological data (relVO2_max_), RStudio [63] was used, based on R Version 4.1.0 [64]. A linear mixed-effects model was applied using lme4 [65]. A random intercept was used to account for individual differences; age and sex were used as covariates. In the model, time was treated as a continuous variable to preserve as much information as possible. Another model was constructed with time treated as a factor, in order to be able to make post-hoc comparisons. Post-hoc tests were performed with emmeans [66], comparing different time points within each group, as well as comparing IG and CG at every time point. *p*-values were adjusted using a multivariate t-distribution to account for all 6 possible comparisons of time points within each group and 4 possible comparisons of both groups at different time points. Results were considered significant at *p* < 0.05. All figures of post-hoc comparisons in this paper were created using ggplot2 [67].

#### 2.4.2. Resting State fMRI Data

A voxelwise linear mixed model approach, as implemented by 3dLMEr [68], was applied to investigate longitudinal changes in FC from each OFC seed to the rest of the brain. In order to diminish the effects of age and sex on the results, they were used as covariates. A random intercept was used to account for individual differences. To avoid artifactual components that might still have persisted up until this point, a grey matter mask was used for the analysis. Multiple comparison corrections were applied using a clusterwise method as implemented by 3dClustSim [69,70]. Group and time interactions were reported at an alpha level <0.05 clusterwise corrected for multiple comparisons and at a voxel level *p* < 0.001.

A significant interaction effect, as assessed by the group difference for the slope effect of time in the linear mixed-effects (LME) model, represents a different profile of FC to the presented region in the IG compared to the CG over the duration of the intervention. A negative (or blue, as shown in the images) effect equals a significantly lower development of FC in the IG compared to the CG, meaning a decrease or a stronger decrease. Another possibility is an increase or a stronger increase in the CG, or a combination of the aforementioned effects. A positive (or red, as visualized in the images) interaction effect equals the opposite, which is a significantly higher development of FC in the IG compared to the CG. Further details on the direction and magnitude of a significant interaction effect were assessed in the post-hoc analysis. In order to present a full picture of the analysis, as proposed by Chen et al. in a recent publication [71], we also searched for trends by lowering the threshold to *p* < 0.001, uncorrected with a cluster size threshold of k = 10. The results were visualized using AFNI’s SUMA tool [72,73].

To perform post-hoc analyses, average beta estimates from a significant cluster were extracted. Using lme4, we constructed a linear mixed model with time as a factor and implemented post-hoc comparisons with R package emmeans [66]. *p*-values were adjusted using the same approach as explained above. The results were considered significant at *p* < 0.05.

#### 2.4.3. Mood Questionnaires

Data from the mood questionnaires were already analyzed in our previous paper [27]; we briefly summarize the results.

#### 2.4.4. Correlations

In the case of significant exercise-related changes in the studied behavioral variables, their association with respective significant changes in OFC FC was assessed using correlation analysis. As the sample size was small, the correlations of the changes (between T0 and T6) were calculated for both groups together, as well as separately in an exploratory approach. Correlations were visualized using R package ggpubr, based on ggplot2 [67], and considered significant at *p* < 0.05.

## 3. Results

### 3.1. Participants/Demographics

Participants’ characteristics, as assessed by the questionnaires and testing at baseline, are presented in the table below (Table 2). The physical characteristics and scores of the initial questionnaires did not yield a significant group difference, as calculated using independent-samples t-tests (or Mann–Whitney U-tests, where necessary) and Fisher’s exact test in the case of sex.

### 3.2. Fitness

**Figure 2:**
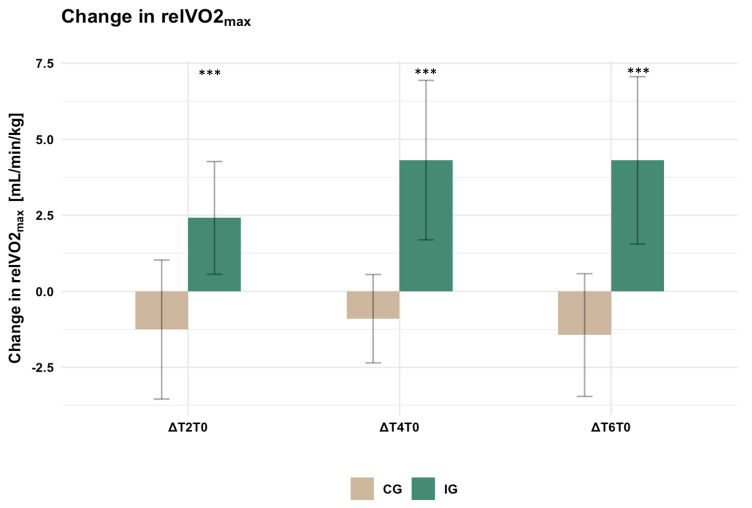
Bar chart representing the changes in relVO2_max_ for the intervention group (IG) and control group (CG) in comparison to baseline measurement (mean ± standard deviation). ΔT2T0: 2 months compared to baseline, ΔT4T0: 4 months compared to baseline, ΔT6T0: 6 months compared to baseline. Significant within-group differences compared to baseline are labeled: *** *p* < 0.001.

As reported in our recent publications [26,27], we detected a significant interaction effect for time and group in the linear mixed model, when assessing relVO2_max_. While the covariate age did not have a significant influence, a significant effect for sex was found, corresponding to male participants generally showing higher relVO2_max_ values compared to females. Post-hoc tests showed a significant increase in relVO2_max_ in the IG for T2 (*p* = 0.001, d = 1.44), T4 (*p* < 0.001, d = 2.57), and T6 (*p* < 0.001, d = 2.60) compared to T0, while no significant change was found in the CG (Figure 2). We did not find any significant difference in relVO2_max_ between the groups at T0, T2, T4, and T6 (see Figure S3).

### 3.3. Seed-to-Whole-Brain Analysis

**Figure 3:**
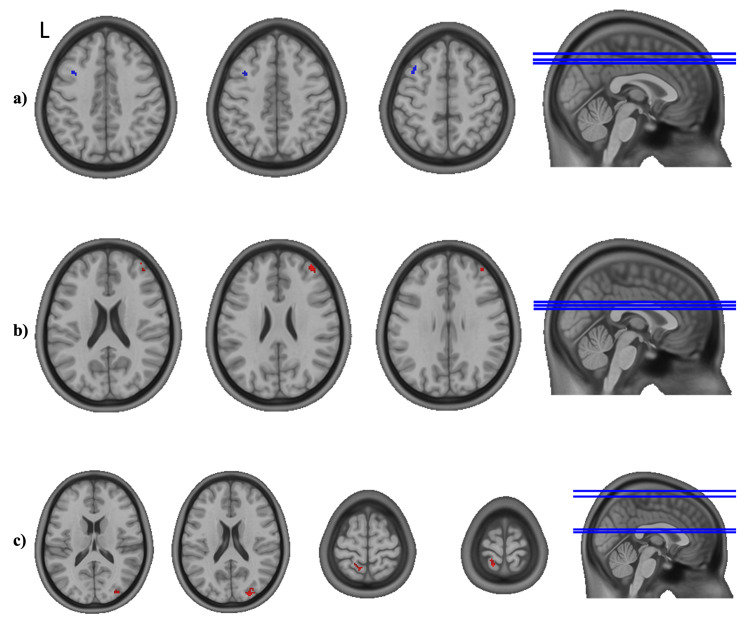
Significant group and time interaction effects from the linear mixed-effects model for the orbitofrontal cortex (OFC) seeds. (**a**) Seed R4 (posterior–lateral right OFC) showing a significant (negative) interaction effect in the left dorsolateral prefrontal cortex. (**b**) Seed R6 (anterior–lateral right OFC) showing a significant (positive) interaction effect in the right middle frontal gyrus. (**c**) Seed L4 (posterior–lateral left OFC) showing significant (positive) interaction effects in the left postcentral gyrus and the right occipital gyrus. The position of each axial slice is illustrated by a blue line in the sagittal plane. Results are shown on the MNI152NLin2009cAsym standard space template [47].

The analysis of the FC showed no significant group difference for the slope effect of time (group and time interaction) for the left and right Seed Regions 1–3 (medial, post-central, and central OFC). For Seed Region 4 on the right side (R4, posterior–lateral right OFC), we detected a significant group and time interaction effect with the left DLPFC (Figure 3a; peak voxel: [−39 19 49], k = 45). For Seed Region 6 on the right side (R6, anterior–lateral right OFC), we found a significant interaction effect of group and time in a small area of the right middle frontal gyrus (MFG) (Figure 3b; peak voxel: [37 51 27], k = 44). Seed Region 4 on the left side (L4, posterior–lateral left OFC) revealed two areas with significant group and time interactions (Figure 3c): one in the left superior postcentral gyrus (peak voxel: [−17 −51 75], k = 55) and one area in the right superior occipital gyrus (peak voxel: [25 −91 19], k = 57). Seed Regions R5 (mid-lateral right OFC), L5 (mid-lateral left OFC), and L6 (anterior–lateral left OFC) did not result in a significant group and time interaction effect. Further details on the direction and magnitude of the significant interaction effects are presented in the post-hoc analysis below. In order to present a full picture of the analysis, trends are visualized in the Supplementary Materials (see Figure S4).

### 3.4. Post-Hoc Analyses

**Figure 4:**
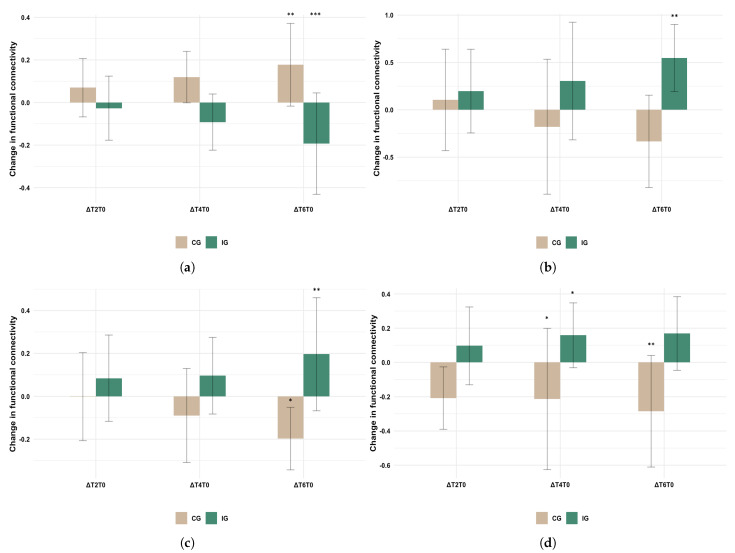
Bar charts created from post-hoc analysis of changes in functional connectivity (FC) in the intervention group (IG) and control group (CG) (mean ± standard deviation). (**a**) FC changes of orbitofrontal cortex (OFC) seed R4 to a cluster in the left dorsolateral prefrontal cortex. (**b**) FC changes of OFC seed R6 to a cluster in the right middle frontal gyrus. (**c**) FC changes of OFC seed L4 to a cluster in the left postcentral gyrus. (**d**) FC changes of OFC seed L4 to a cluster in the right occipital gyrus. ΔT2T0: 2 months compared to baseline, ΔT4T0: 4 months compared to baseline, ΔT6T0: 6 months compared to baseline. Significant within-group differences compared to baseline are labeled: * *p* < 0.05, ** *p* < 0.01, *** *p* < 0.001.

Post-hoc analyses on the extracted average values from significant clusters, performed in R, showed that the change in FC from R4 to the DLPFC was significant from baseline to T6, for both the IG (*p* < 0.001, d = −1.63) and the CG (*p* = 0.006, d = 1.52); while for the CG, an increase could be observed, the FC of the IG decreased (Figure 4a). For the change in FC from R6 to the MFG, the increase from T0 to T6 in the IG was significant (Figure 4b; *p* = 0.002, d = 1.31). As for the change in FC from L4 to the area in the postcentral gyrus, the change in T6 compared to T0 was again significant in both the CG (decrease; *p* = 0.014, d = −1.39) and IG (increase; *p* = 0.001, d = 1.36) (see Figure 4c). The difference in FC to the occipital gyrus at T6 compared to T0, on the other hand, was only significant within the CG (decrease; *p* = 0.004, d = −1.6) (see Figure 4d). We tested for group differences at all time points. The results and corresponding figures can be found in the Supplementary Materials (see Figure S5a–d). Furthermore, we also tested for change score differences between the groups (interaction contrasts). The detailed results of all post-hoc contrasts can be found in the Supplementary Materials (see Tables S1 and S2).

### 3.5. Mood Questionnaires

There was no significant group and time interaction effect for the STAI state or the positive and negative scales of the PANAS. A detailed analysis can be found in our previous published study [27].

### 3.6. Correlation Analyses

As the sample size was small and our analysis of the affective scale questionnaires (STAI state, PANAS) did not result in a significant group and time interaction effect, we decided not to search for a correlation between the observed changes in the FC and the outcomes of outcomes of questionnaires.

**Figure 5:**
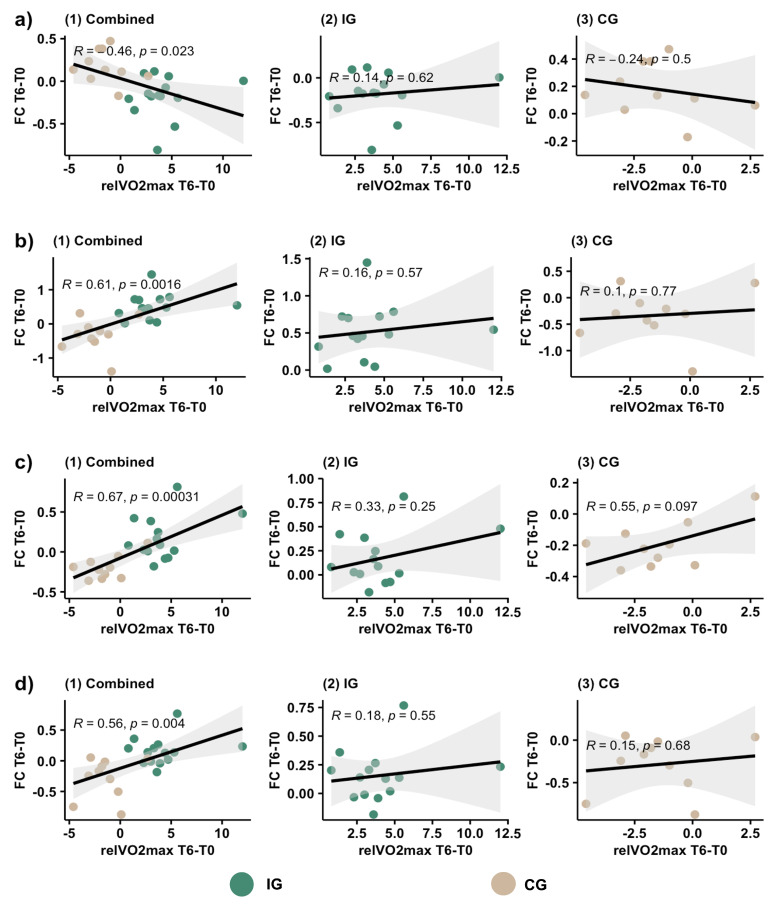
Scatter plot and correlation between changes in relVO2_max_ and changes in functional connectivity (FC) for the intervention group (IG) and control group (CG). From left to right: (1) Correlation of both groups combined. (2) Correlation for IG only. (3) Correlation for CG only. (**a**) Correlation of changes in relVO2_max_ with changes in FC from orbitofrontal cortex (OFC) seed R4 to the cluster in the left dorsolateral prefrontal cortex. (**b**) Correlation of changes in relVO2_max_ with changes in FC from OFC seed R6 to the cluster in the right middle frontal gyrus. (**c**) Correlation of changes in relVO2_max_ with changes in FC from OFC seed L4 to the cluster in the left postcentral gyrus. (**d**) Correlation of changes in relVO2_max_ with changes in FC from OFC seed L4 to the cluster in the right occipital gyrus. relVO2_max_ T6–T0: difference in relVO2_max_ between measurement after six months (T6) and baseline (T0), FC T6–T0: difference in FC between measurement after six months and baseline; calculated by subtracting the value at T0 from the value at T6.

We examined the correlation between the changes (T6 compared to T0) in relVO2_max_ and in FC for both groups combined, as well as for the groups separately. While we did find a significant correlation between the change in FC and the change in relVO2_max_ in the overall correlation for R4→DLPFC (Figure 5a; R = −0.46, *p* = 0.023), R6→MFG (Figure 5b; R = 0.61, *p* = 0.0016), L4→postcentral gyrus (Figure 5c; R = 0.67, *p* = 0.00031), and L4→occipital gyrus (Figure 5d; R = 0.56, *p* = 0.004), the correlation did not appear significant when exploring both groups separately.

## 4. Discussion

In our study, we observed differential changes in FC for the subdivisions of the OFC after a longitudinal exercise intervention in young healthy adults. We found distinct effects of regular PA on the FC profiles of the lateral parts of the OFC: We observed a significant group and time interaction effect for the FC between the lateral Seed Region 4 on the right side with the left DLPFC. Moreover, Seed Region 6 on the right side showed a group and time interaction effect with the right MFG, while Seed Region 4 on the left side revealed a significant group and time interaction with the left postcentral gyrus and right occipital gyrus. These results suggest that the FC profiles of lateral regions of the OFC were differentially altered by the longitudinal exercise intervention in these healthy young adults. Furthermore, the within- and cross-hemispheric short- and long-range FC changes suggest a possible modulation of cortical networks associated with lateral OFC subcomponents.

We observed a significant decrease in the FC of the posterior–lateral right OFC to the DLPFC in the IG. While statistically significant for the left DLPFC, this can also be found as a trend for the right DLPFC. Kahnt et al. [11] reported the lateral OFC to be connected to the DLPFC, lateral PFC, dorsomedial PFC, inferior parietal cortex, and lateral temporal cortex. More connections of the lateral OFC have been presented by other authors [2]. Another study reported an increase in FC between the lateral OFC and the right DLPFC in patients with major depression [8]. In our study, we did not observe any significant changes in mood questionnaires between the CG and IG, but a trend time effect in positive PANAS [27]. We speculate that this was due to the study population consisting of healthy subjects who did not show high variance in the PANAS and STAI, where a ceiling or floor effect is likely to be found and significant changes in mood might not be expected. In contrast to the introspective questionnaires, FC changes might be more sensitive towards showing the effects of the regular physical exercise. As this decrease in FC observed in the IG over the time of our exercise study is the opposite to the findings reported in patients with MD, it supports the hypothesis that some treatments for depression act by reducing the FC of the lateral OFC [2]. The same author also suggested that another approach for treatments of depression might be to increase the FC of the medial OFC, which could, however, not be confirmed in the current dataset.

Furthermore, we observed an increase in the FC from the anterior–lateral right OFC (R6) to the MFG. That area is part of the frontoparietal network and has been described to be important for executive function [74]. While there is not much literature regarding the FC to the OFC, the reduced FC of the right MFG to the right intermediate OFC was found in patients with schizophrenia in another study, which was based on a different parcellation of the OFC [75]. The FC of the OFC to the left MFG has also been found inversely correlated with anger in patients with post-traumatic stress disorder [76]. While it was not significant in our analysis, an increased FC to the left MFG did show up as a trend. Even though this does not provide robust evidence of the importance of the FC from the OFC to the MFG in psychiatric disorders, this could be an aspect to further examine in future studies.

Another finding showed a weaker negative FC from the posterior–lateral left OFC (L4) to the left superior postcentral gyrus in the IG and a stronger negative FC in the CG. The postcentral gyrus receives somatosensory input from the body, and when looking at the somatotopic representation, the data in our study are compatible with significant clusters representing the trunk area. In this context, we found only two previous works reporting a change in the FC of the OFC to an area of the postcentral gyrus: Research on children with externalizing disorders found that adolescents with a positive family history of substance use disorders had a stronger negative FC between the left lateral OFC and the right postcentral gyrus [77]. Another study reported a decreased FC of the left OFC to several areas of the (bilateral) postcentral gyrus in patients with schizophrenia [57]. While this again points towards the relevance of that connection in psychiatric disorders, there is not enough evidence to make further assumptions.

Finally, we observed a stronger negative FC (starting from a slightly positive FC at baseline) from L4 to the right occipital gyrus/visual cortex in the CG. Even though the link between the visual cortex and the OFC has been well established [78], the exact meaning of this finding remains unclear. Zou et al. [79] found that the OFC, precuneus, superior temporal gyrus, and visual cortex act as important hubs and reported decreased long-range FC density in patients with MD. This could indicate that, while regular PA helps prevent affective disorders, being sedentary might facilitate their onset. A recent study concluded that sedentary behavior is a risk factor for anxiety and depressive symptoms, and even short periods of moderate to vigorous activity could reduce this risk [80]. However, there is currently a lack of FC data to support this claim and understand the underlying neuronal mechanisms. Therefore, further research is needed to grasp the changes in OFC FC induced by psychiatric disorders and those influenced by regular PA.

Correlation analyses of FC changes and relVO2_max_ development showed a significant correlation overall, but the effect was not observable when performing an exploratory analysis in the IG and CG separately. Although a possible reason may be the small sample size, resulting in inconsistent results, a visual analysis of the plotted values illustrates that both groups differed significantly, but no apparent connection of relVO2_max_ improvement and change in FC can be identified within the groups themselves. This indicates that adhering to a regular scheme of moderate exercise appears to be an important factor for the modulation of OFC connectivity, whilst the achieved amount of fitness improvement in the IG did not strictly correlate with FC changes.

A limitation of the present study was the small number of subjects, in particular in the CG, which limited the statistical power. Accordingly, the robustness of the current findings needs to be confirmed in future studies, preferentially with larger samples. In contrast to a previous study on healthy young individuals, which found decreases in mood disturbance in the IG [81], we did not find significant changes in our mood questionnaires. As we already discussed previously, the questionnaires used were optimized for the assessment of current mood and, therefore, are more susceptible to acute changes [27]. Even with questionnaires targeted specifically at sustained mood, however, the results depend on the subjects’ ability and willingness to accurately reflect and describe their emotions. Possible limitations include difficulties in understanding the emotional vocabulary, emotions outside of phenomenal consciousness, social desirability, and measurement reactivity [82]. An unbiased approach towards overcoming this limitation might be to include behavioral paradigms, e.g., tasks including face processing, affective bias, or emotive images [83]. While the fMRI sequence parameters and planning were not optimized for the specific purpose of data acquisition in orbitofrontal regions, the systematic signal dropout for the distorting-corrected EPI images due to susceptibility artifacts was restricted to the circumscribed subgenual area of the OFC, not affecting the seed coordinates for our data analysis (Figure S2). Meanwhile, future studies may further improve the robustness of the data collection by using OFC-tailored acquisition protocols.

## 5. Conclusions

The changes in FC from the posterior–lateral right OFC to the DLPFC observed in the IG were the opposite to what has been reported for patients with MD, hence emphasizing previous findings that regular PA is beneficial for patients with affective disorders and could even play a role in preventing them. Significant changes in the FC to other brain regions (FC from the anterior–lateral right OFC to the MFG and from the posterior–lateral left OFC to the left superior postcentral gyrus and the right visual cortex) also support the hypothesis that these might be beneficial for maintaining brain homeostasis. The findings remain descriptive, given that no changes in mood questionnaires could be found in this healthy sample. Therefore, possible associations with mood parameters remain unclear, and further research will be needed to identify the importance of the observed FC changes with regard to mood, and, ultimately, its role in psychiatric disorders. To summarize, the importance of the present FC changes in the OFC as the outcome parameter of PA interventions should be validated in larger samples and extended to clinical cohorts to validate the clinical relevance of these FC changes.

## Figures and Tables

**Table 1:** MNI coordinates of the seeds used in the analysis. Based on Kahnt et al. [11].

	Left Hemisphere (L)			Right Hemisphere (R)		
Seed No.	x	y	z	x	y	z
1	−7	49	−12	6	47	−13
2	−19	35	−19	16	28	−19
3	−26	36	−13	37	32	−8
4	−43	30	−11	40	31	−14
5	−42	46	−8	31	39	−12
6	−28	57	−8	28	54	−8

**Table 2:** Demographics from the RUNSTUD study.

Variables	IG (N = 18)	CG (N = 10)	*p*-Value
Sex (male/female)	7/11	6/4	0.433 ^b^
Age (years)	23.9 ± 3.9	23.7 ± 4.2	0.879
Height (cm)	174 ± 12.1	177 ± 7.9	0.441
Weight (kg)	69.9 ± 15.1	71.2 ± 14.1	0.649 ^a^
BMI (kg/m^2^)	23.1 ± 3.7	22.7 ± 3.6	0.762
HR_max_ (1/min)	198 ± 7.6	201 ± 8.5	0.468
relVO2_max_ (mL/min/kg)	38.5 ± 3.4	41.7 ± 7.5	0.232
Education (years)	16.3 ± 3.1	15.8 ± 3.1	0.781 ^a^
BDI	2.6 ± 3.4	1.4 ± 1.5	0.704 ^a^
STAI trait	33.9 ± 9.3	31.4 ± 6.1	0.624 ^a^
FTND	0.2 ± 0.9	0.0 ± 0.0	n/a ^c^
WST IQ	107 ± 9.9	107 ± 8.8	0.937
EHI_LQ	74.2 ± 16.2	79.5 ± 13.3	0.390

Values are the mean ± the standard deviation. *p*-values were calculated using an independent t-test (or ^a^ Mann–Whitney U-test). ^b^ *p*-value for sex (categorical) was calculated using Fisher’s exact test. ^c^ The whole sample contained only one smoker. BMI: body mass index, HR_max_: maximal heart rate during graded exercise task, relVO2_max_: highest 30 s average oxygen uptake per kg body mass, BDI: Beck Depression Inventory, STAI trait: trait scale of the State-Trait Anxiety Inventory, FTND: Fagerström Test for Nicotine Dependence, WST IQ: verbal intelligence quotient of the German vocabulary test, EHI_LQ: Laterality Quotient of the Edinburgh-Handedness-Inventory, IG: intervention group, CG: control group.

## Data Availability

The data presented in this study are available upon reasonable request from the corresponding author. The data are not publicly available due to privacy and/or ethical restrictions.

## Supplementary Materials

The following Supporting Information can be downloaded at: https://www.mdpi.com/article/10.3390/healthcare11050689/s1: Figure S1: Flow diagram (reprint). Figure S2: Functional connectivity of the orbitofrontal cortex in the RUNSTUD dataset; Figure S3: Bar chart with absolute values of relVO2_max_; Figure S4: Trends from seed-to-whole-brain-analysis; Figure S5: Bar charts with absolute values of functional connectivity; Table S1: Results of post-hoc comparisons based on fitted LME model and observed data for change scores; Table S2: Results of interaction contrasts based on fitted LME model and observed data for change score differences between the groups.

## Abbreviations

The following abbreviations are used in this manuscript:

BDI	Beck Depression Inventory
BOLD	Blood-oxygenation-level-dependent
CG	Control group
CSF	Cerebrospinal fluid
DLPFC	Dorsolateral prefrontal cortex
EPI	Echo-planar imaging
FC	Functional connectivity
IG	Intervention group
MD	Major depression
MFG	Middle frontal gyrus
OFC	Orbitofrontal cortex
PANAS	Positive and Negative Affect Schedule
PA	Physical activity
relVO2_max_	Maximal oxygen uptake (mL/min/kg)
fMRI	Functional magnetic resonance imaging
ROI	Region of interest
rsfMRI	Resting state fMRI
STAI	State-Trait-Anxiety Inventory
WM	White matter
