# Supplementary material for: Physical Activity Alters Functional Connectivity of Orbitofrontal Cortex Subdivisions in Healthy Young Adults: A Longitudinal fMRI Study
Source: Healthcare (Basel). 2023 Feb 26;11(5):689. doi: 10.3390/healthcare11050689 (PMC10001322; doi:10.3390/healthcare11050689)
Supplement: Supplementary file 1 [file healthcare-11-00689-s001.zip › healthcare-2148912-supplementary.pdf]

Supplementary Information

Physical Activity Alters Functional Connectivity of Orbitofrontal Cortex Subdivisions in Healthy Young Adults: A Longitudinal fMRI Study

J. Claus, N. Upadhyay, A. Maurer, J. Klein, L. Scheef, M. Daamen, J. A. Martin, R. Stirnberg, A. Radbruch, U. Attenberger, T. Stöcker, H. Boecker*

* Corresponding author. E-Mail: Henning.Boecker@ukbonn.de

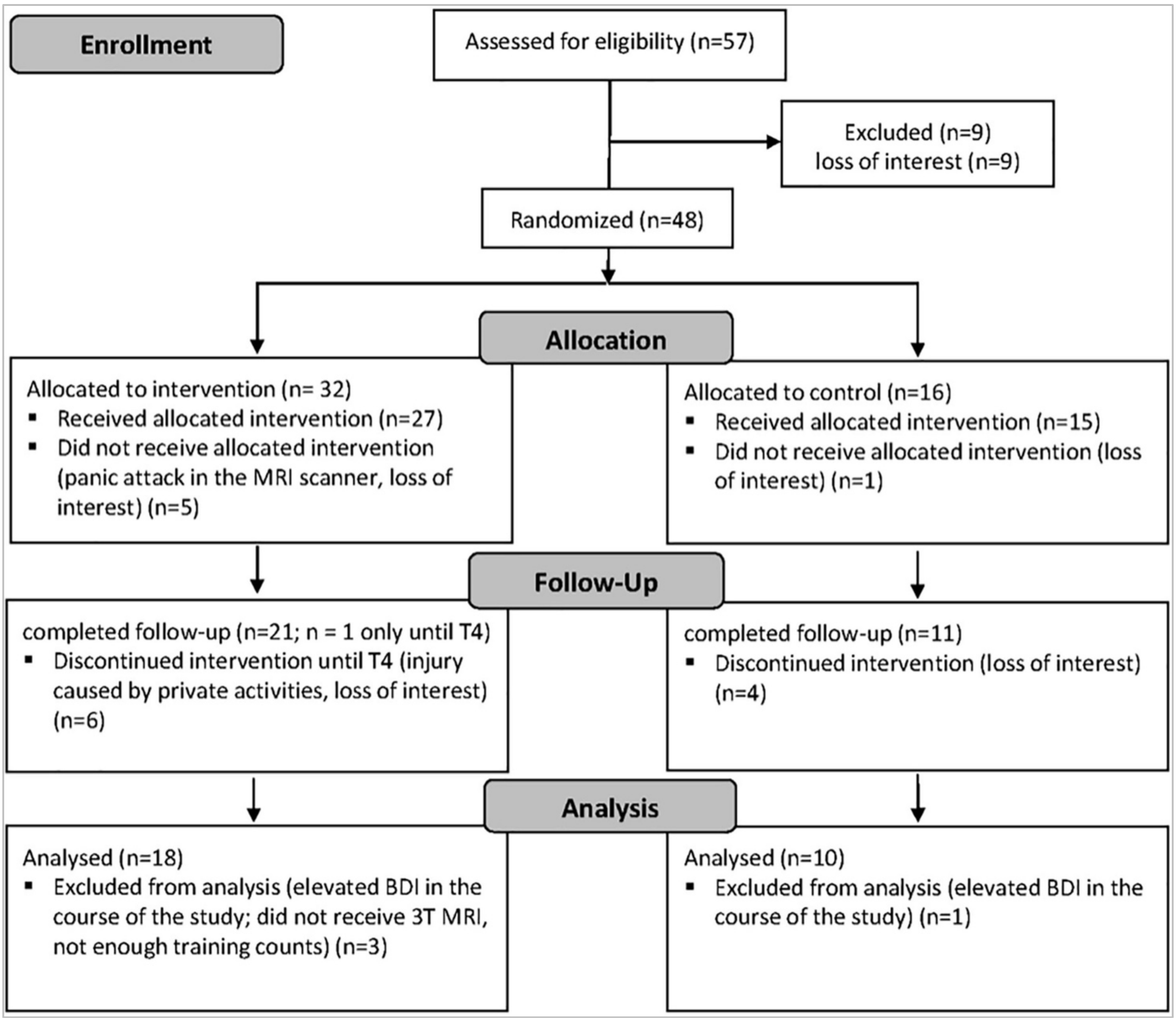

Figure S1. Flow diagram. Reprinted from *Upadhyay, N.; Schörkmaier, T.; Maurer, A.; Claus, J.; Scheef, L.; Daamen, M.; Martin, J.A.; Stirnberg, R.; Radbruch, A.; Attenberger, U.; et al. Regional Cortical Perfusion Increases Induced by a 6-Month Endurance Training in Young Sedentary Adults. Front. Aging Neurosci. 2022, 14, 951022. Licensed under CC BY 4.0 (https://creativecommons.org/licenses/by/4.0/).*

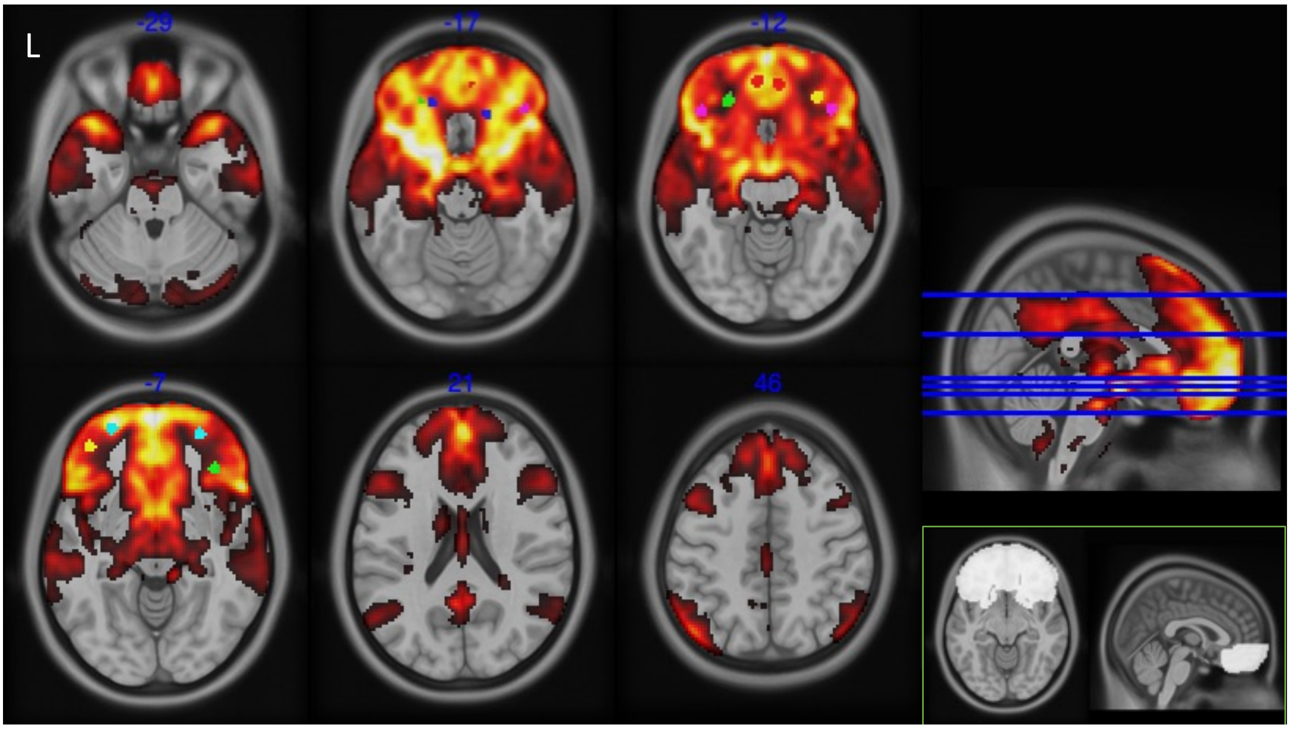

Figure S2. Functional connectivity (FC) of the orbitofrontal cortex (OFC) in the RUNSTUD data set. The seed of the bilateral OFC (displayed in the bottom right corner) was generated based on the Harvard-Oxford atlas. FC topography maps were calculated using dual regression (FSL) on the denoised data. Results were then averaged across all participants and sessions and overlayed on MNI152NLin2009cAsym standard space template using MRIcron; thresholded at 0.1 for visual purposes. To demonstrate sufficient coverage of the OFC, the detailed seeds used in this present work are overlayed on top. Colouring of the seeds is analogous to figure 1 in this paper: 1=red, 2=blue, 3=green, 4=violet, 5=yellow, 6=cyan.

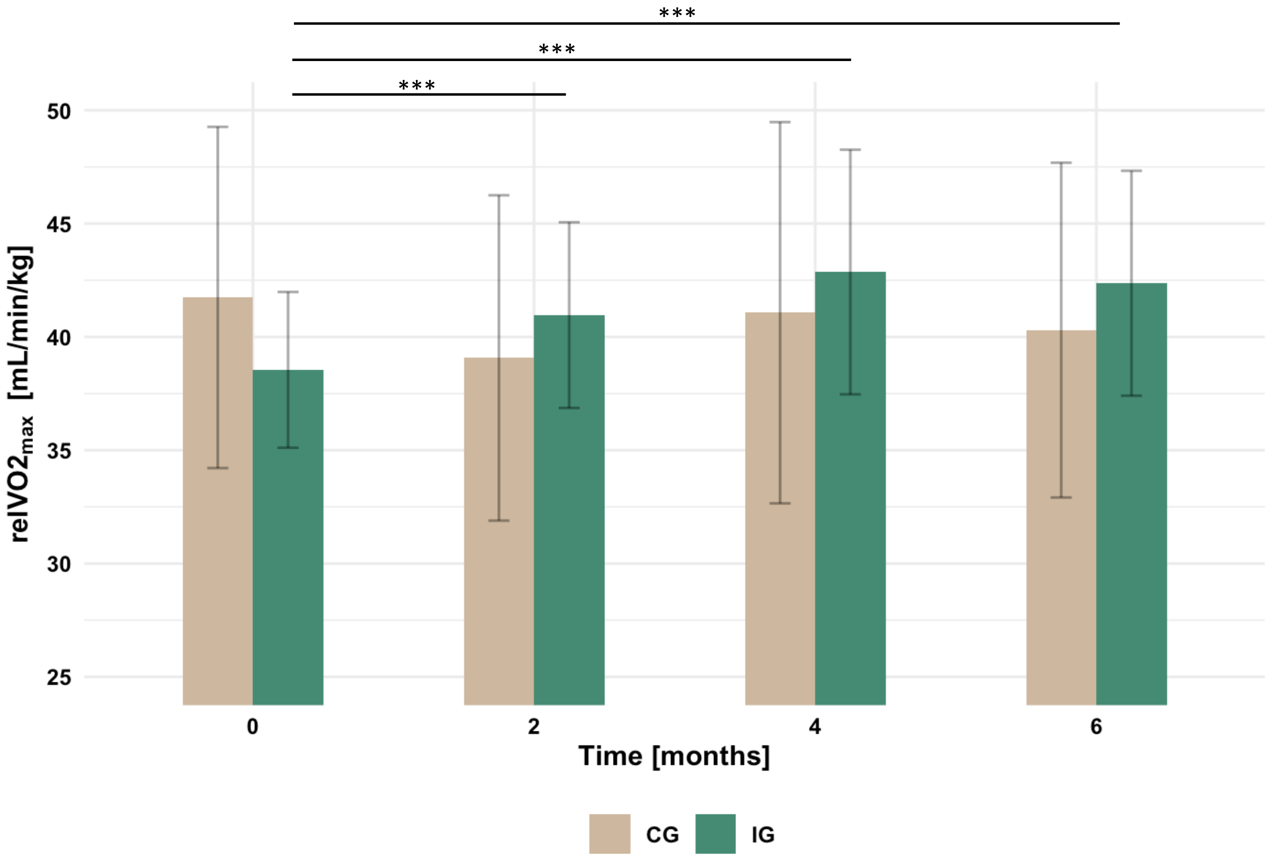

Figure S3. Bar chart with absolute values of relVO2_{max} in intervention group (IG) and control group (CG) for each timepoint (mean ± standard deviation). Significant within-group differences compared to baseline and between-group differences are labelled: *** $p < 0.001$.

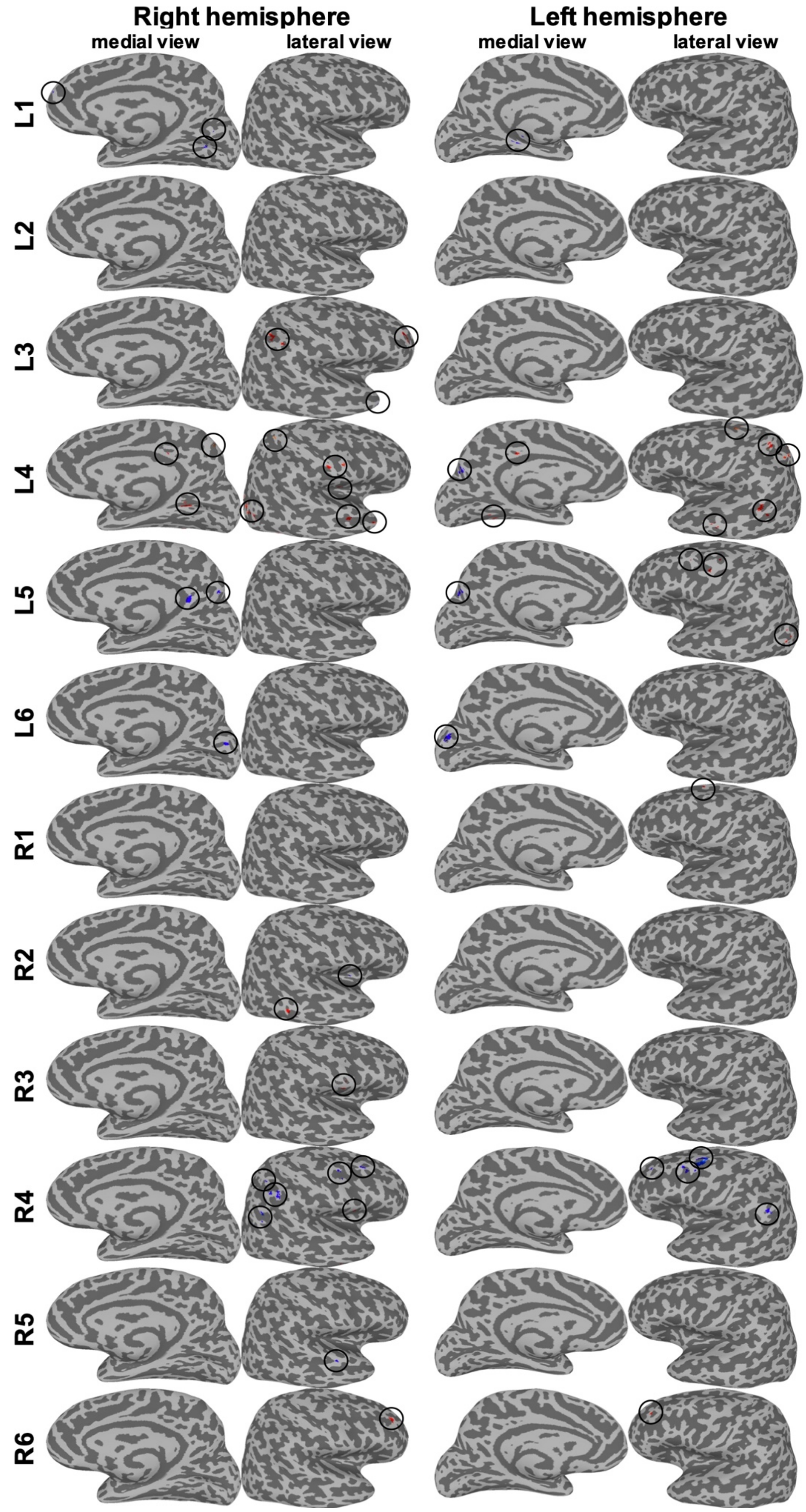

Figure S4. Trends from seed-to-whole-brain-analysis (with k=10 and $p<0.001$ uncorrected) for each seed 1-6 in the left (L) and right (R) orbitofrontal cortex. 1: medial, 2: post-central, 3: central, 4: posterior-lateral, 5: mid-lateral, 6: anterior-lateral. Created using AFNI Surface Mapper (SUMA).

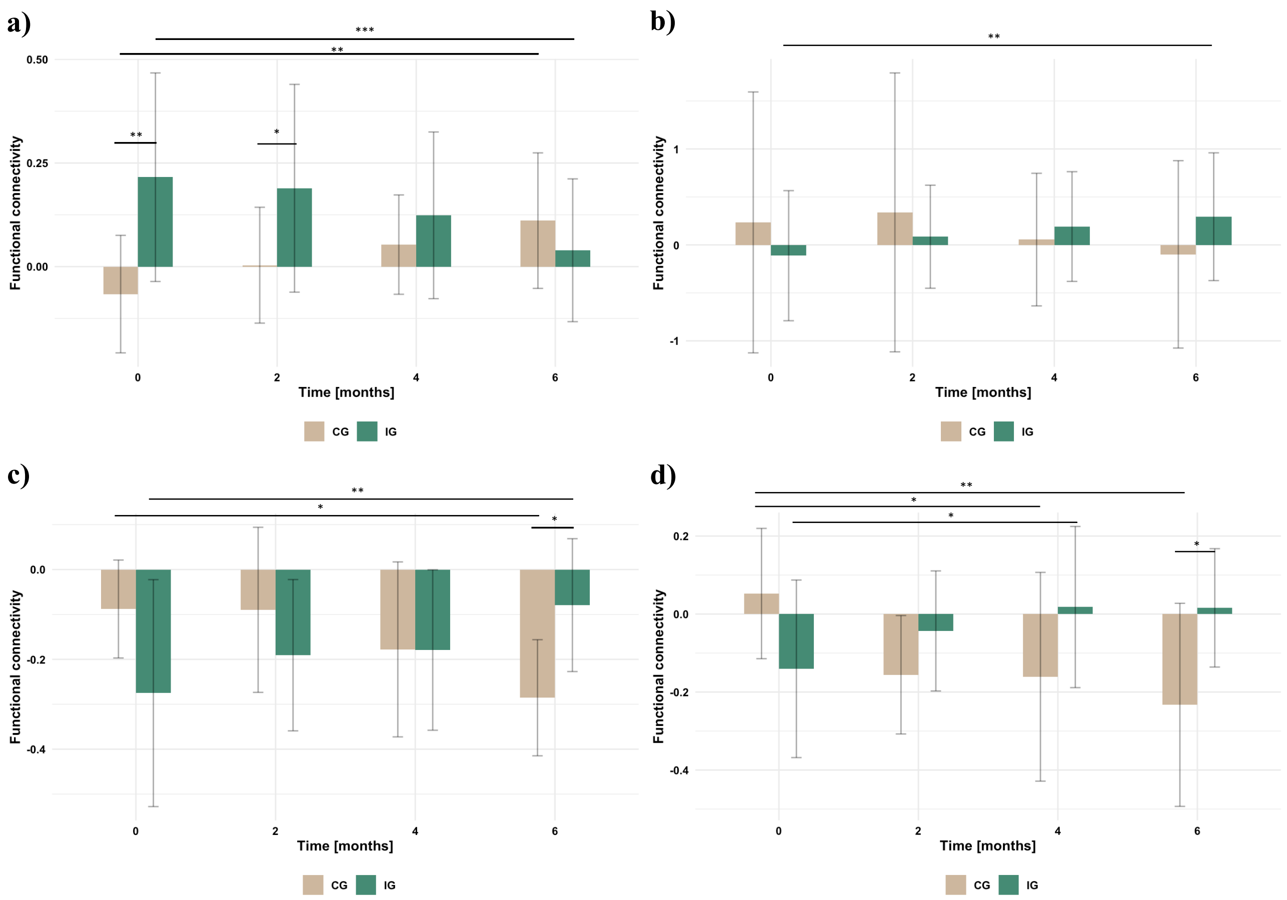

Figure S5. Bar charts with absolute values of functional connectivity (FC) in intervention group (IG) and control group (CG); and group differences (mean ± standard deviation). Created based on post-hoc analysis. **(a)** FC of orbitofrontal cortex (OFC) seed R4 to the cluster in the left dorsolateral prefrontal cortex **(b)** FC of OFC seed R6 to the cluster in the right middle frontal gyrus **(c)** FC of OFC seed L4 to the cluster in the left postcentral gyrus **(d)** FC of OFC seed L4 to the cluster in the right occipital gyrus. Significant within-group differences compared to baseline and between-group differences are labelled: * $p < 0.05$, ** $p < 0.01$, *** $p < 0.001$.

	Group	Contrast	Observed data		Results of post-hoc comparisons based on fitted LME model					
			Mean	SE	Estimate	SEE	p value	Cohen's d	CI (95%)	
**relVO2$_{max}$**	CG	T2-T0	-1.260	0.864	-0.917	0.855	0.707	-0.545	-1.592	0.502
		T4-T0	-0.900	0.484	-0.648	0.782	0.840	-0.385	-1.342	0.572
		T6-T0	-1.440	0.638	-1.440	0.752	0.231	-0.856	-1.784	0.071
	IG	T2-T0	2.410	0.463	2.415	0.595	0.001***	1.436	0.677	2.196
		T4-T0	4.320	0.655	4.316	0.595	<.0001***	2.567	1.738	3.396
		T6-T0	4.310	0.711	4.371	0.607	<.0001***	2.599	1.754	3.444
**R4→DLPFC**	CG	T2-T0	0.070	0.043	0.070	0.052	0.542	0.599	-0.308	1.505
		T4-T0	0.119	0.038	0.119	0.052	0.109	1.025	0.122	1.929
		T6-T0	0.177	0.061	0.177	0.052	0.006**	1.521	0.618	2.425
	IG	T2-T0	-0.027	0.036	-0.027	0.039	0.902	-0.229	-0.902	0.445
		T4-T0	-0.092	0.031	-0.092	0.039	0.092	-0.789	-1.472	-0.107
		T6-T0	-0.193	0.060	-0.190	0.040	0.0001***	-1.633	-2.371	-0.895
**R6→MFG**	CG	T2-T0	0.104	0.169	0.104	0.163	0.918	0.287	-0.624	1.199
		T4-T0	-0.179	0.225	-0.179	0.163	0.690	-0.493	-1.406	0.421
		T6-T0	-0.333	0.154	-0.333	0.163	0.180	-0.916	-1.830	-0.002
	IG	T2-T0	0.198	0.104	0.198	0.121	0.368	0.544	-0.136	1.223
		T4-T0	0.304	0.146	0.304	0.121	0.067	0.835	0.156	1.514
		T6-T0	0.547	0.089	0.475	0.126	0.002**	1.305	0.595	2.015
**L4→postcent.**	CG	T2-T0	-0.002	0.065	-0.002	0.064	1.000	-0.013	-0.906	0.881
		T4-T0	-0.090	0.069	-0.090	0.064	0.493	-0.633	-1.531	0.265
		T6-T0	-0.198	0.046	-0.198	0.064	0.014*	-1.392	-2.307	-0.476
	IG	T2-T0	0.084	0.047	0.084	0.047	0.289	0.595	-0.077	1.266
		T4-T0	0.096	0.042	0.096	0.047	0.187	0.676	0.003	1.349
		T6-T0	0.196	0.066	0.193	0.049	0.001**	1.356	0.638	2.074
**L4→occip.**	CG	T2-T0	-0.208	0.058	-0.208	0.080	0.054	-1.160	-2.053	-0.267
		T4-T0	-0.213	0.130	-0.213	0.080	0.046*	-1.189	-2.082	-0.296
		T6-T0	-0.285	0.103	-0.285	0.080	0.004**	-1.590	-2.490	-0.689
	IG	T2-T0	0.097	0.054	0.097	0.060	0.372	0.541	-0.126	1.208
		T4-T0	0.158	0.045	0.158	0.060	0.047*	0.882	0.212	1.553
		T6-T0	0.169	0.054	0.156	0.062	0.066	0.868	0.174	1.563

Table S1. Results of post-hoc comparisons based on fitted LME model using R package emmeans, and observed data for change scores. IG: Intervention group, CG: Control group, T2-T0: 2 months compared to baseline, T4-T0: 4 months compared to baseline, T6-T0: 6 months compared to baseline, R4→DLPFC: Functional connectivity (FC) changes of orbitofrontal cortex (OFC) seed R4 to a cluster in the left dorsolateral prefrontal cortex, R6→MFG: FC changes of OFC seed R6 to a cluster in the right middle frontal gyrus, L4→postcent.: FC changes of OFC seed L4 to a cluster in the left postcentral gyrus, L4→occip.: FC changes of OFC seed L4 to a cluster in the right occipital gyrus, SE: Standard error, SEE: Standard error of the estimate, CI (95%): 95% confidence interval for Cohen's d. * $p < 0.05$, ** $p < 0.01$, *** $p < 0.001$.

	Change score difference		Results of post-hoc comparisons based on fitted LME model			
	Contrast	mean	estimate	SEE	p value	Cohen’s d
**relVO2max**	IG-CG (T2-T0)	3.670	3.332	1.041	0.011*	1.981
	IG-CG (T4-T0)	5.220	4.964	0.982	<.0001***	2.952
	IG-CG (T6-T0)	5.750	5.81	0.967	<.0001***	3.456
**R4→DLPFC**	IG-CG (T2-T0)	-0.096	-0.096	0.065	0.453	-0.827
	IG-CG (T4-T0)	-1.038	-0.211	0.065	0.009**	-1.815
	IG-CG (T6-T0)	-0.370	-0.367	0.066	<.0001***	-3.154
**R6→MFG**	IG-CG (T2-T0)	0.094	0.093	0.203	0.968	0.256
	IG-CG (T4-T0)	0.483	0.483	0.203	0.089	1.328
	IG-CG (T6-T0)	0.880	0.808	0.206	0.001**	2.221
**L4→postcent.**	IG-CG (T2-T0)	0.086	0.086	0.079	0.697	0.607
	IG-CG (T4-T0)	0.186	0.186	0.079	0.096	1.309
	IG-CG (T6-T0)	0.394	0.390	0.080	<.0001***	2.748
**L4→occip.**	IG-CG (T2-T0)	0.305	0.305	0.100	0.017*	1.701
	IG-CG (T4-T0)	0.371	0.372	0.100	0.002**	2.071
	IG-CG (T6-T0)	0.454	0.441	0.101	0.0002***	2.458

Table S2. Results of interaction contrasts based on fitted LME model using R package emmeans, and observed data for change score differences between the groups. IG: Intervention group, CG: Control group, IG-CG (T2-T0): Change score difference between IG and CG at 2 months, IG-CG (T4-T0): Change score difference between IG and CG at 4 months, IG-CG (T6-T0): Change score difference between IG and CG at 6 months, R4→DLPFC: Functional connectivity (FC) changes of orbitofrontal cortex (OFC) seed R4 to a cluster in the left dorsolateral prefrontal cortex, R6→MFG: FC changes of OFC seed R6 to a cluster in the right middle frontal gyrus, L4→postcent.: FC changes of OFC seed L4 to a cluster in the left postcentral gyrus, L4→occip.: FC changes of OFC seed L4 to a cluster in the right occipital gyrus, SE: Standard error, SEE: Standard error of the estimate. * $p < 0.05$, ** $p < 0.01$, *** $p < 0.001$.
